# Cervical ectopic pregnancy of 11 amenorrhea weeks: clinical image

**DOI:** 10.1093/omcr/omae191

**Published:** 2025-02-22

**Authors:** Aboubakr Benjilany, Jaouad Kouach

**Affiliations:** Department of obstetrics and gynecology, Military hospital of instruction Mohamed V, Rabat 10000, Morocco; Department of obstetrics and gynecology, Military hospital of instruction Mohamed V, Rabat 10000, Morocco

**Keywords:** sexual and reproductive health, radiology, nuclear medicine and medical imaging, paediatrics, emergency medicine

Cervical ectopic pregnancy (CEP) is a rare location of ectopic pregnancy in which implantation and nidation occur in the endocervical canal [1]. Several management strategies have been reported, medical treatment whenever possible (Methotrexate, Potassium chloride, etc.). Radical surgical treatment, however, is indicated in the presence of hemodynamic instability or failure of medical treatment [2]. Selective embolization of the uterine arteries constitutes a new therapeutic approach, which is of great interest in young patients and/or those wishing to preserve their fertility [3].

We reported a case of a 30-year-old patient, nulliparous, with a history of endouterine curettage for spontaneous hemorrhagic abortion of 8 amenorrhea weeks, 2 years ago. Consulted our emergency department with moderate pelvic pain 2 weeks old, minimal reddish metrorrhagia and 11 weeks’ amenorrhea. General examination revealed a hemodynamically stable patient. Gynecological examination found a soft, bulging, shortened cervix with a partially open external cervical ostium. Conception product is perceptible through it. Endovaginal ultrasound (Fig. 1A) exposed an empty uterus with decidualized endometrium (asterisk), an evolving intra-cervical pregnancy of 11 weeks’ amenorrhea with a closed internal cervical ostium (arrow). Magnetic resonance imaging (MRI) (Fig. 1B) confirmed the endocervical location of the pregnancy with thinning of the posterior cervical stroma (arrow). The diagnosis of cervical ectopic pregnancy -extremely rare- was retained. Given the high risk of maternal haemorrhage, the decision was to perform a selective uterine artery embolization as a preventive and possibly therapeutic measure. Pelvic angiography (Fig. 2A) revealed that the ectopic pregnancy was principally supplied by the right uterine artery (arrow). Post-embolization imaging showed satisfactory bilateral occlusion of uterine arterial flow (Fig. 2B: arrows). Additional medical treatment with methotrexate and surgical curettage (Fig. 3) were successfully established, with no clinical and biological blood loss. The post-operative course was without abnormalities.

**Figure 1:**
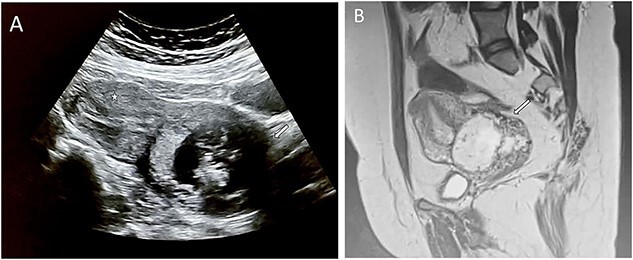
**(**A) Endovaginal ultrasound showing an evolving intracervical pregnancy of 11 amenorrhea weeks (arrow). (B) Magnetic resonance imaging confirming the endocervical location of the pregnancy with thinning of the posterior cervical stroma (arrow).

**Figure 2:**
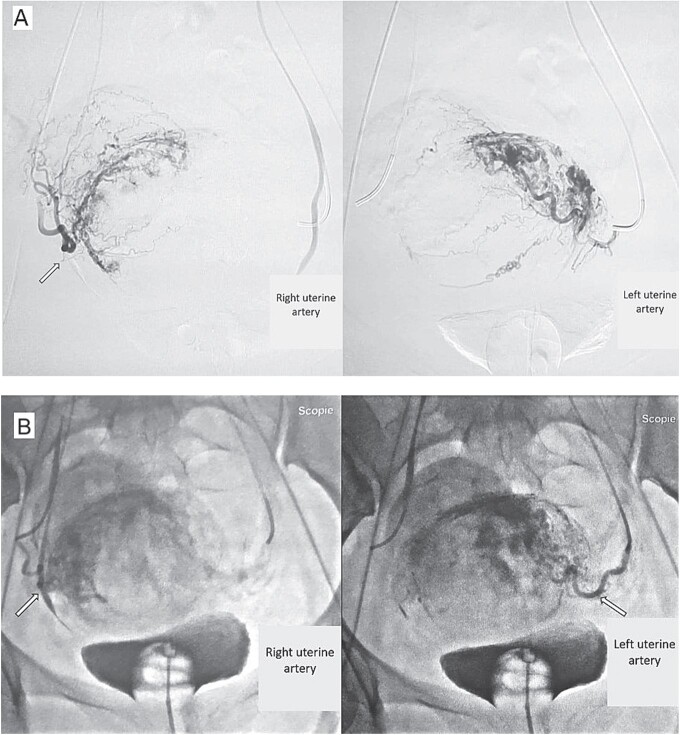
**(**A) Pre-embolization pelvic angiography showing vascular mapping of the uterine arteries. (B) Post-embolization pelvic angiography showing satisfactory bilateral occlusion of the uterine arterial flow.

**Figure 3:**
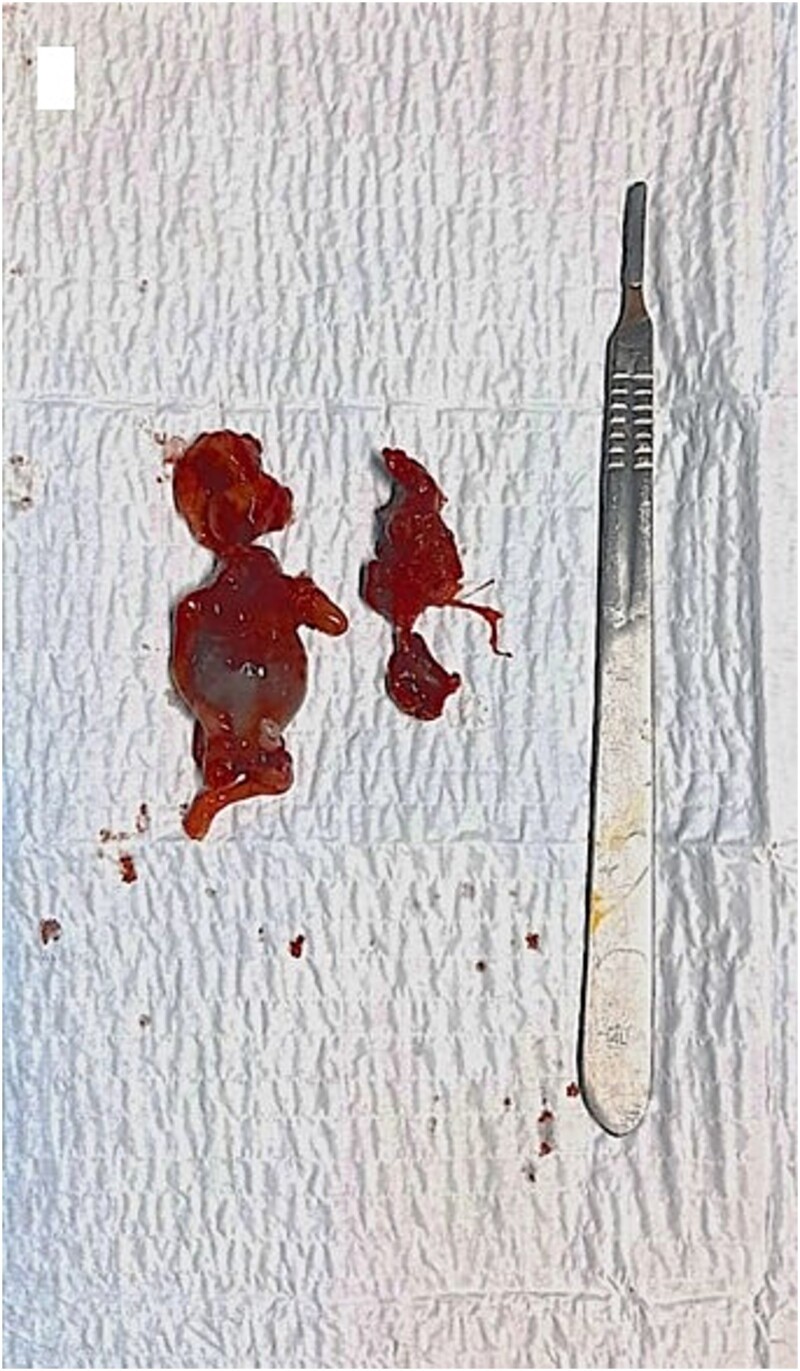
Clinical appearance of the product of conception in the immediate post-curettage.

CEP is a very rare pathology, with a high hemorrhagic risk. Selective embolization of uterine arteries can be used as part of a minimally invasive treatment strategy of CEP. Whenever patient’s status allows it, technical platform and qualified personnel are available, obstetricians should consider this new approach in consultation with interventional radiologists.

## Consent For Publication

Written consent has been obtained from the patient for the publication of this clinical report and images.

## Guarantor

Aboubakr Benjilany.
